# Computerized Modeling of Adenosine Triphosphate, Adenosine Triarsenate and Adenosine Trivanadate 

**DOI:** 10.3390/molecules17089489

**Published:** 2012-08-08

**Authors:** Valter A. Nascimento, Petr Melnikov, Lourdes Z. Z. Consolo

**Affiliations:** School of Medicine of the Federal, University of Mato Grosso do Sul/UFMS, Caixa Postal 549, 79070-900 Campo Grande/MS, Brazil; Email: petrmelnikov@yahoo.com (P.M.); lzzanoni@yahoo.com.br (L.Z.Z.C.)

**Keywords:** ATP, arsenic, arsenate, arsenic metabolism, adenosine triarsenate, adenosine trivanadate, computerized molecular models

## Abstract

Computerized molecular models of adenosine triphosphate, adenosine tri-arsenate and adenosine trivanadate have been generated using the molecular mechanics technique. The analysis of structural parameters indicated that, at least theoretically, adenosine triarsenate is a realistic candidate for replacement of adenosine triphosphate in biochemical pathways. On the contrary, the structural arrangement of the inorganic segment of adenosine trivanadate does not seem to be capable of withstanding a swift hydrolytical splitting in aqueous milieu. It was shown that the universal force field as implemented in Gaussian software packages is an appropriate tool for the optimization of less-common bioactive compositions.

## 1. Introduction

Nature has chosen phosphorus as a foundation for the key biological molecules such as adenosine triphosphate (ATP) and nucleic acids. This choice is based on the fact that hydrolysis of these anionic species is thermodynamically favorable, but occurs only slowly. Earthly metabolism relies on the accumulation of phosphate derivatives and the controlled catalysis of transfer of inorganic phosphates between biochemically active species [1]. The phosphate ion acts as an essential participant in maintaining the structure of DNA and RNA and as a basic building block of energy metabolism, where it is a principal form of energy available to cells. 

It is known that submitochondrial particles from beef heart can produce an arsenic-containing analog of ATP if they are provided with arsenate [2]. That gives rise to the supposition that there may exist a parallel biochemical medium, rich in arsenic, where phosphorus is substituted for its closest analog in the Periodic Table. As a matter of fact, a bacterium found in the arsenic-filled waters of a Californian lake is supposed to substitute arsenic for phosphorus to sustain its growth [3,4], although arsenate had been known as a possible modifier of ATP and ADP for a long time [5,6]. Indeed, the interatomic distances and interbond angles in tripolyphosphate and triarsenate ions are rather similar (Table 1), with the exception of the angles P-O_b_-P and As-O_b_-As between the corresponding tetrahedra. 

**Table 1:** Structural Parameters of Tripolyphosphate [7] and Triarsenate [8] Ions.

	Tripolyions			
	P_3_O_10_^5−^		As_3_O_10_^5−^	
*Distances (Å)*	P-O_b_ *	1.52	As-O_b_ *	1.67
	P OH	1.61	As OH	1.74
	O-P-O	96	O-As-O	93
*Angles (°)*	P-O_b_-P	156	As-O_b_-As	120

* Bridging oxygen.

In view of the structural similarities between orthophosphate and orthovanadate ions (V-O bond length 1.66 Å in tetrahedral coordination), vanadium is another possible candidate for phosphorus replacement. The discovery that vanadium as the inorganic polyanion inhibits Na, K-ATPase was unexpected and intriguing [9]. Nevertheless, these findings never seemed of practical importance, although the idea of partial substitution as the reason for ATP inhibition had been put forward a long time ago [6]. 

**Figure 1:**
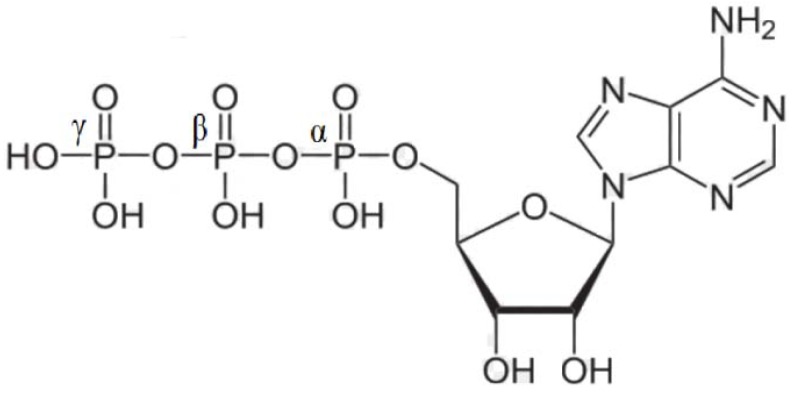
Schematic representation of the protonated form of ATP.

In any case, the phosphorus atoms at the positions α, β and γ of the scheme (Figure 1) can be totally or partially replaced by arsenic. A recently published review hypothesizes that ancient biochemical systems, analogous to but distinct from those known today, could have utilized arsenate in place of phosphate in an equivalent biological role [10]. The problem, however, consists in the experimentally established fact that polyarsenate units are more easily hydrolyzed than polyphosphates and may lack the qualities needed for effective action.

Several attempts have been made to describe the breakage of the P-O_b_-P linkage and to understand the underlying hydrolytic mechanisms. The approaches range from simple molecular orbital calculations to contemporary quantum mechanical techniques. Recently, the Car-Perinello molecular dynamics method has been used to study ATP hydrolysis in water and the results compared to the experimentally determined structural parameters [11]. No single crystals and, consequently, no structural data are available so far for the arsenic substituted form AT-As, although, there are grounds to suppose that these closely related compounds can be isostructural.

In this context, the use of computerized modeling techniques may be appropriate in evaluating the missing parameters for AT-As in order to compare the structural arrangements of ATP and AT-As in detail. The aim of the present work is to perform the simulations of ATP and AT-As structural arrangements using such modern software in order to elucidate the nature of the possible differences in their biochemical activity. The analysis focuses on the P (As)-Ob-P (As) moiety and the bond angles. On the other hand, vanadate’s ability to mimic phosphate in a transition state configuration does not permit one to reject outright this element as a biological effector [12], so in order to explore a broader structural approach, additional calculations have been carried out for the vanadium-containing moiety, AT-V.

## 2. Molecular Modeling

The structures resulting from substitution of arsenic for phosphorus in ATP were simulated using the universal force field potential as implemented in the Gaussian 03 [13] and Gaussview 4.1.2 [14] software packages. This is the most promising force field available at this time, widely used for systems containing elements other than carbon, hydrogen oxygen and nitrogen. It contains the energy terms for bond stretching, angle bending, dihedral torsion, Van der Waals and electrostatic potential [15]. The geometry optimization was carried out in Cartesian coordinates using the Berny optimization algorithm, and adjusting the parameters until a stationary point on the potential surface was found. That means that for a small displacement the energy does not change within a certain amount, and the placements are successfully converged. Angles and interatomic distances were evaluated by using special features of the program. 

Molecular mechanics calculations are deceptively simple to perform. However, in order to trust the results, it is advisable to simulate primarily a closely related compound with solidly established structural characteristics. Generally, these are obtained on the basis of X-ray studies for solids or NMR RMN for liquids, as was recently done in a work dedicated to cysteine and selenocysteine [16]. A reasonable agreement between simulated and experimental data would constitute a sufficient guarantee for a proposed model to be capable of existing as an independent unit. In the present work we chose the deprotonated form of ATP as a reference compound to be simulated; at the same time, the X-ray data published on sodium salts and inorganic derivatives of tripolyphosphate anion were employed for comparison. 

## 3. Results and Discussion

**Figure 2:**
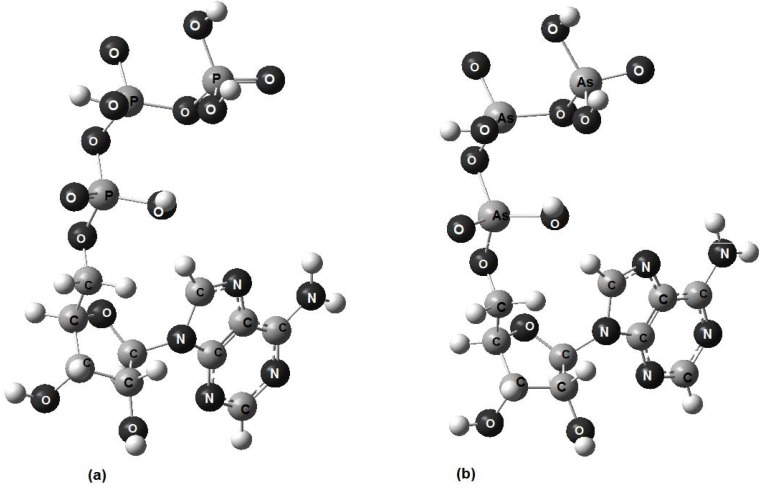
(**a**) A stereoview of the model representing protonated form of ATP. The angle between the adenine ring and the plane of sheet is approximately 50°; (**b**) A similar view of the arsenic-containing protonated form, ATAs.

**Figure 3:**
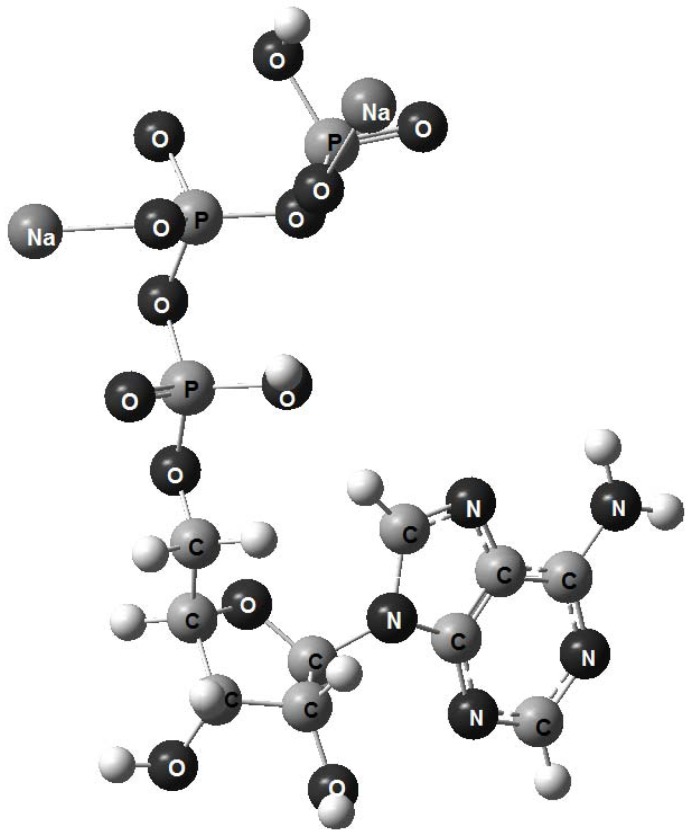
A stereoview of the model representing ATP sodium salt.

**Figure 4:**
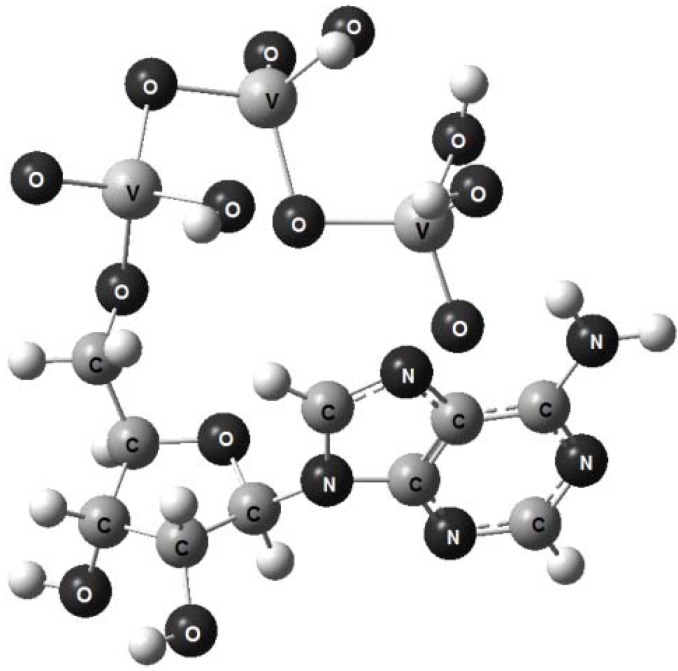
A stereoview of the model representing vanadium-containing protonated form, ATV.

The models obtained using the molecular mechanics technique are shown in Figure 2, Figure 3, Figure 4; the small balls, left unlabelled, represent hydrogen atoms. A stereo view was chosen in order to center attention on the tripolyphosphate section, which enables the molecule to adapt its conformation to the surrounding environmental conditions [15]. In all three molecules this chain is not extended in shape, but rather folds back towards the adenine base, just as was found for crystalline disodium ATP salt [17]. In a real solid the folding of tripolyphosphate tail gives rise to the left and right-handed helixes, but for an isolated molecule the existence of stereoisomers is of no importance. 

As can be seen, the optimized graphic representations for ATP, ATAs and Na2ATP are strikingly similar, so the differences, if any, can be perceived only by comparison of the main structural parameters: the interatomic distances and bond angles.

According to Table 2, the distances P = O, P–O and H…O in our models are practically the same, coinciding satisfactorily not only with the previously calculated values, but also with results obtained from the X-ray diffractograms for related compounds. The distances within the bridges P-O_b_ are also consistent with the parameters determined by other methods. As for the bonds C-O_b_ and H…O, they are identical for all models, including arsenic and vanadium-containing compounds. Ribose and adenine rings remain unaffected by changes in their inorganic part.

**Table 2:** Structural parameters obtained for the adenosine triphosphate models, as compared with calculated [7] * and experimentally established [13] ** values.

Parameters	Compounds				Published data	
ATP	Na_2_ATP	AT-As	AT-V	calc.	exper.
*Distances*						
E *** = O	1.51	1.49	1.64	1.75	1.50	1.50
E-O	1.68/1.69	1.60	1.83	1.96	1.57	1.50
E-O_b_	1.70	1.60	1.85	1.96	1.58/1.65	1.61
C-O_b_	1.40	1.42	1.40	1.39	1.48	1.42
H. . .O	0.99	0.97	0.99	0.99	0.98/1.04	-
*Angles*						
E-O_b_-E	107.6	112.6/114.6	105.7	104.6	119.5	125.6
E-O_b_-C	108.6	124.1	107.8	105.4	120	122.9

* Density functional method for ATP; ** X-ray data for Na_2_ATP; *** *E* stands for phosphorus, arsenic or vanadium in tetrahedral coordination.

Meanwhile, the key distances As-O and As-O_b_ are higher, though not excessively so, in comparison with ATP: only 1.64 and 1.83 Å compared to 1.50 and 1.60/1.69 Å for the phosphorus-containing compositions, that is with a difference of about 10%. It is true that the hydrolysis mechanism is very complex, including dissociative and associative reactions and involving magnesium ions in the presence of a system of extensively branched hydrogen bonds. Nevertheless, all conditions being equal, the As-O_b_ bond within AT-As cannot be considered so weak that a cleavage could occur without any great difficulty. These data further corroborate the aforementioned hypothesis that adenosine triarsenate, under certain circumstances, can serve the same functions as adenosine triphosphate. 

In contrast, the V-O_b_ distances are considerably more extended: 1.96 Å, that is, twice as much as for As-O_b_. This is a clear indication that vanadium compound must be less stable in aqueous solutions and show a propensity to rapid hydrolysis.

As for the angles E-O_b_-E and E-O_b_-C, they do not differ substantially in size in any of the three adenosine triphosphates. At the same time, the angles obtained in this work for Na_2_ATP and those calculated by Car-Perinello technique for the same compound are identical. However, the former are larger due to significant deformations which occur as a result of sodium coordination with phosphate oxygens and adenine ring nitrogen. 

## 4. Conclusions

1. Computerized molecular models of adenosine triphosphate, adenosine triarsenate and adenosine trivanadate have been proposed.
2. The analysis of structural parameters shows that ATP and ATAs are stereochemically equivalent.
3. The structural arrangement of adenosine trivanadate does not seem to be capable of withstanding a swift hydrolytical splitting in aqueous milieu.
4. The universal force field as implemented in the Gaussian software packages is an appropriate tool for the optimization of less-common bioactive compositions.
